# Combined Phacoemulsification, Goniosynechialysis and Ab Interno Trabeculectomy in Primary Angle-closure Glaucoma: Long-term Results

**DOI:** 10.7150/ijms.103795

**Published:** 2025-01-01

**Authors:** Fengrui Yang, Yao Ma, Zhiqiao Liang, Kun Lv, Kangyi Yang, Huijuan Wu

**Affiliations:** 1 Department of Ophthalmology, Peking University People's Hospital, No. 11 Xizhimen South Street, Xicheng District, Beijing, China, 100044.; 2 Eye diseases and Optometry Institute, No. 11 Xizhimen South Street, Xicheng District, Beijing, China, 100044.; 3 Beijing Key Laboratory of Diagnosis and Therapy of Retinal and Choroid Diseases, No. 11 Xizhimen South Street, Xicheng District, Beijing, China, 100044.; 4 College of Optometry, Peking University Health Science Center, No. 11 Xizhimen South Street, Xicheng District, Beijing, China, 100044.

**Keywords:** primary angle-closure glaucoma, ab-interno trabeculectomy, Trabectome, Kahook Dual Blade

## Abstract

**Objective:** This research was designed to evaluate the efficacy and safety of ab-interno trabeculectomy (Trabectome and Kahook Dual Blade) combined with phacoemulsification, intraocular lens implantation, and goniosynechialysis in eyes with primary angle-closure glaucoma.

**Methods:** A total of 47 patients were included in the study and all the patients received the combined surgery. Intraocular pressure, anti-glaucoma medications, best-corrected visual acuity, and the number of peripheral anterior synechiae quadrants were recorded at baseline and at various time points after surgery.

**Results:** Intraocular pressure decreased significantly from 20.93 ± 6.53 mmHg preoperatively to 15.09 ± 3.63 mmHg (*P* < 0.001) at 36 months. The number of glaucoma medications was significantly reduced from 2.45 ± 1.18 preoperatively to 1.25 ± 1.55 (*P* = 0.001) at 36 months. The success rate of the combined surgery was 90.0% at 36 months. The decrease of intraocular pressure exhibited a positive correlation with the baseline intraocular pressure (*P* < 0.001), while the reduction in the number of glaucoma medications was positively correlated with the baseline number of glaucoma medications (*P* < 0.001). Best-corrected visual acuity improved from 0.39 ± 0.29 to 0.48 ± 0.34 at 1 month (*P* = 0.005). There were no vision‑threatening complications intraoperatively or postoperatively.

**Conclusion:** The combined surgery has been proven to be effective and safe for patients with primary angle-closure glaucoma in the long term, suggesting that combined surgery may be beneficial for patients with primary angle-closure glaucoma, especially those with long-term and extensive peripheral anterior synechiae.

## Introduction

Primary angle-closure glaucoma (PACG) is a condition characterized by elevated intraocular pressure (IOP) caused by angle closure, which can lead to irreversible damage to the optic nerve. According to International Society for Geographical and Epidemiological Ophthalmology (ISGEO), PACG is an eye with an occludable drainage angle and features indicating that trabecular obstruction by the peripheral iris, together with the evidence of glaucoma 1. The main risk factors for PACG include small cornea, shallow anterior chamber, thick lens, anterior lens position, and short axial length 2. The global prevalence of PACG is 0.6% 3, while the prevalence in China is 1.40-1.41% 4. PACG has become an important cause of blindness in Asia and worldwide 5. With the population rapidly aging, the glaucoma burden caused by PACG in China is expected to increase significantly.

Minimally invasive or microinvasive glaucoma surgery (MIGS) is increasingly used for the treatment of PACG 6. Ab-interno trabeculectomy (AIT) using Trabectome or Kahook Dual Blade (KDB) is an important surgical procedure that improves aqueous outflow through the Schlemm canal (SC) 7, 8. However, potential complications such as shallow anterior chamber, choroidal detachment, hypotonous maculopathy, and cataract development should not be overlooked 9. Although cataract surgery alone can reduce IOP by deepening the crowded anterior chamber, its success rate is not satisfactory 10-12. The combination of cataract surgery with goniosynechialysis (GSL) has been proven to effectively reduce IOP and the need for antiglaucoma medications 13. AIT offers several advantages over traditional filtering procedures as it selectively removes the trabecular meshwork (TM) and SC without damaging the remaining components of the outflow system, thus avoiding the complications associated with filtering blebs. Trabectome is a specific type of AIT that effectively removes the TM and inner wall of the SC to reduce IOP, with a low risk of shallow anterior chamber, choroidal detachment, and hypotony maculopathy 14. A previous study by Wang Y *et al.* demonstrated the efficacy and safety of phacoemulsification, intraocular lens (IOL) implantation, GSL and Trabectome in patients with PACG 15. KDB is another specialized instrument used in AIT. AIT with KDB combined with phacoemulsification leads to statistically and clinically significant reductions in both IOP and the use of antiglaucoma medications in eyes with PACG 16-19, while also enhancing best-corrected visual acuity (BCVA) 20. This report presents long-term follow-up data of patients with PACG who were treated with phacoemulsification, IOL implantation, GSL, and AIT (Trabectome or KDB). The aim of this study was to evaluate the efficacy and safety of AIT using KDB and Trabectome when combined with phacoemulsification, IOL implantation, and GSL in patients with PACG. The study also aimed to provide a more comprehensive and long-term assessment of the surgical outcomes while investigating the factors that may influence the success of these procedures.

## Methods

This prospective study was approved by the ethics committee of Peking University People's Hospital and adhered to the principles of the Declaration of Helsinki. Written informed consent was obtained from all participants. Patients were enrolled in the study if they met the following baseline criteria: 1) Synechial angle closure greater than 2 quadrants, as demonstrated on gonioscopy and ultrasound biomicroscopy (UBM); 2) IOP greater than 21 mmHg or normal IOP with the use of at least one anti-glaucoma medication; 3) Glaucomatous optic disk damage (focal or diffuse neuroretinal rim thinning, localized notching, or nerve fiber layer defects) and corresponding visual field (VF) defects; 4) The presence of visually significant cataract. Patients were excluded from the study based on the following criteria: 1) A history of prior intraocular surgery, except for laser peripheral iridotomy, laser iridoplasty, and anterior chamber paracentesis; 2) Previous ocular trauma; 3) Primary open angle glaucoma (POAG) or secondary glaucoma; 4) Other ocular lesions 1, 12, 21.

Only one eye of each patient was included in the research. All patients underwent a comprehensive ophthalmologic examination before surgery, which included the following assessments: BCVA (Snellen visual chart), IOP (Goldmann applanation tonometry), slit-lamp examination, and VF perimetry (Swedish Interactive Threshold Algorithm [SITA] 24-2 test of the Humphrey visual field analyzer 750i, Carl Zeiss Meditec, Dublin, CA). Gonioscopy and UBM (Aviso, Quantel Medical, Inc., Bozeman, MT, USA) were performed to determine the extent of peripheral anterior synechiae (PAS). PAS was defined as a region of irido-trabecular contact that could not be opened by indentation gonioscopy. The UBM examination divided the peripheral iris into four 90-degree quadrants and reported the condition of the anterior chamber angle in each quadrant.

The type of surgery and any intraoperative complications were documented. The following data were collected at 1 day, 1 week, and 1, 3, 6, 12, 24, and 36 months postoperatively: BCVA, IOP, postoperative complications, and the number of IOP-lowering medications used by each patient. In this study, a successful surgery was defined as IOP < 21mmHg with or without IOP-lowering medications 15. Patients who underwent a second anti-glaucoma operation (excluding laser peripheral iridotomy and laser iridoplasty) were immediately classified as unsuccessful.

All surgeries were performed by the same skilled surgeon under topical anesthesia. Standard phacoemulsification and IOL implantation were performed through clear corneal incisions. GSL was performed with a circumferential application of viscoelastic (1ml, Bausch & Lomb, Shandong, CN). With the assistance of an intraoperative surgical gonioscope (Ocular Instrument, Inc., US), a blunt cyclodialysis spatula was gently pressed against the peripheral iris to apply retrograde pressure on the iris and visualize the TM as clearly as possible, facilitating the AIT portion of the procedure.

For Trabectome surgery, the temporal clear corneal incision was enlarged to 1.7 mm, and the Trabectome single-use handpiece with the irrigation-aspiration (I/A) system (Neomedix Inc., Tustin, USA) was inserted into the nasal anterior chamber. The TM and inner wall of the SC were removed over a 90°-120° range using a power of 0.8 W.

In trabeculectomy with KDB, the KDB instrument was introduced through the temporal incision. For the right eye, a new temporal clear corneal incision was made in addition to the primary two, guiding the instrument to the nasal angle under direct gonioscopy. The tip of the instrument was inserted through the TM into the SC and advanced for several clock hours, excising a narrow strip of the TM. During a second pass in the opposite direction, more clock hours of TM were removed, leading to a total TM excision range of approximately 120 degrees per eye.

Reflux hemorrhage from SC upon withdrawal of the handpiece served as a marker of success for both methods. The viscoelastic substance was then removed using the I/A system. Postoperative care included a standard course of antimicrobial prophylaxis for 1 week and anti-inflammatory therapy tapered over 4 weeks. Patients typically received levofloxacin eye drops (5 ml: 24.4 mg, Santen Pharmaceutical Co., Ltd., Osaka, Japan) four times a day in the first week after surgery and tobramycin/dexamethasone eye drops (5 ml: 15mg/5mg, SA Alcon-Couvreur N.V., Puurs, Belgium) four times a day after surgery, gradually reducing the frequency over four weeks. The use of IOP-lowering medications was determined based on postoperative IOP levels, with postoperative inflammation and intraocular hemorrhage also considered in medication application.

Data were analyzed using SPSS 26.0 software (Chicago, USA). Arithmetic means and changes from the baseline are presented as ± standard deviation (SD). The distribution of the data was validated using the Kolmogorov-Smirnov test. A paired samples t-test was conducted to compare pre- and post-surgical findings, including IOP, antiglaucoma medications, BCVA, and number of PAS quadrants. The success of the procedure was analyzed using Kaplan-Meier analysis. Variables hypothesized to be associated with a reduction in IOP or a decrease in the number of glaucoma medications were analyzed through univariate linear regression. These variables included age, baseline IOP, the number of preoperative glaucoma medications, and the number of preoperative PAS quadrants. A *P*-value of less than 0.05 was deemed statistically significant.

## Results

Patients with PACG and visually significant cataracts were recruited from the ophthalmology department at a university-affiliated hospital. All patients required surgical intervention to control IOP. Baseline characteristics of all patients are presented in Table 1. A total of 47 Chinese patients, with a mean age of 65.68 ± 8.56 years, were included in the study. The mean follow-up time for all the patients was 40.96 ± 22.71 months. Out of the 47 patients, 22 (46.8%) were male, and 25 (53.2%) were female. All patients underwent phacoemulsification, IOL implantation, GSL, and AIT. Forty patients underwent Trabectome surgery, while seven received trabeculectomy with KDB.

**Figure 1:**
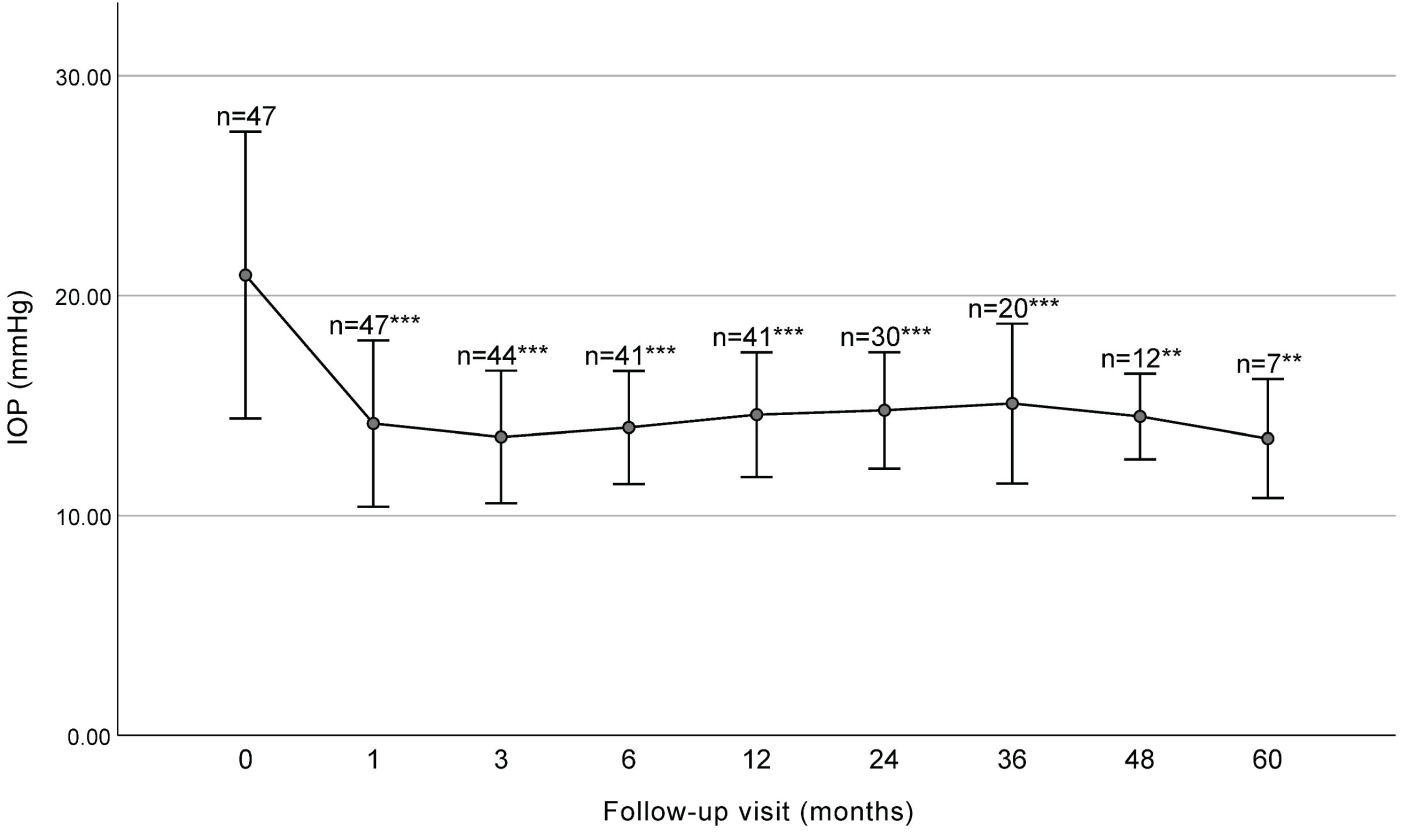
IOP before surgery and at each follow up visit (with SD bars). The IOP significantly decreased at each follow-up visit before 36 months. IOP, intraocular pressure. *** P < 0.001, ** P < 0.01.

The mean IOP at baseline and each follow-up visit is presented in Figure 1 and Table 2. The mean IOP decreased significantly from 20.93 ± 6.53 mmHg preoperatively to 14.78 ± 2.64 mmHg at 2 years (*P*<0.001) and 15.09 ± 3.63 mmHg at 3 years (*P*<0.001) postoperatively. Significant decreases in IOP were observed at 1, 3, 6, 12, 24, 36, 48, and 60 months (*P* < 0.001).

**Figure 2:**
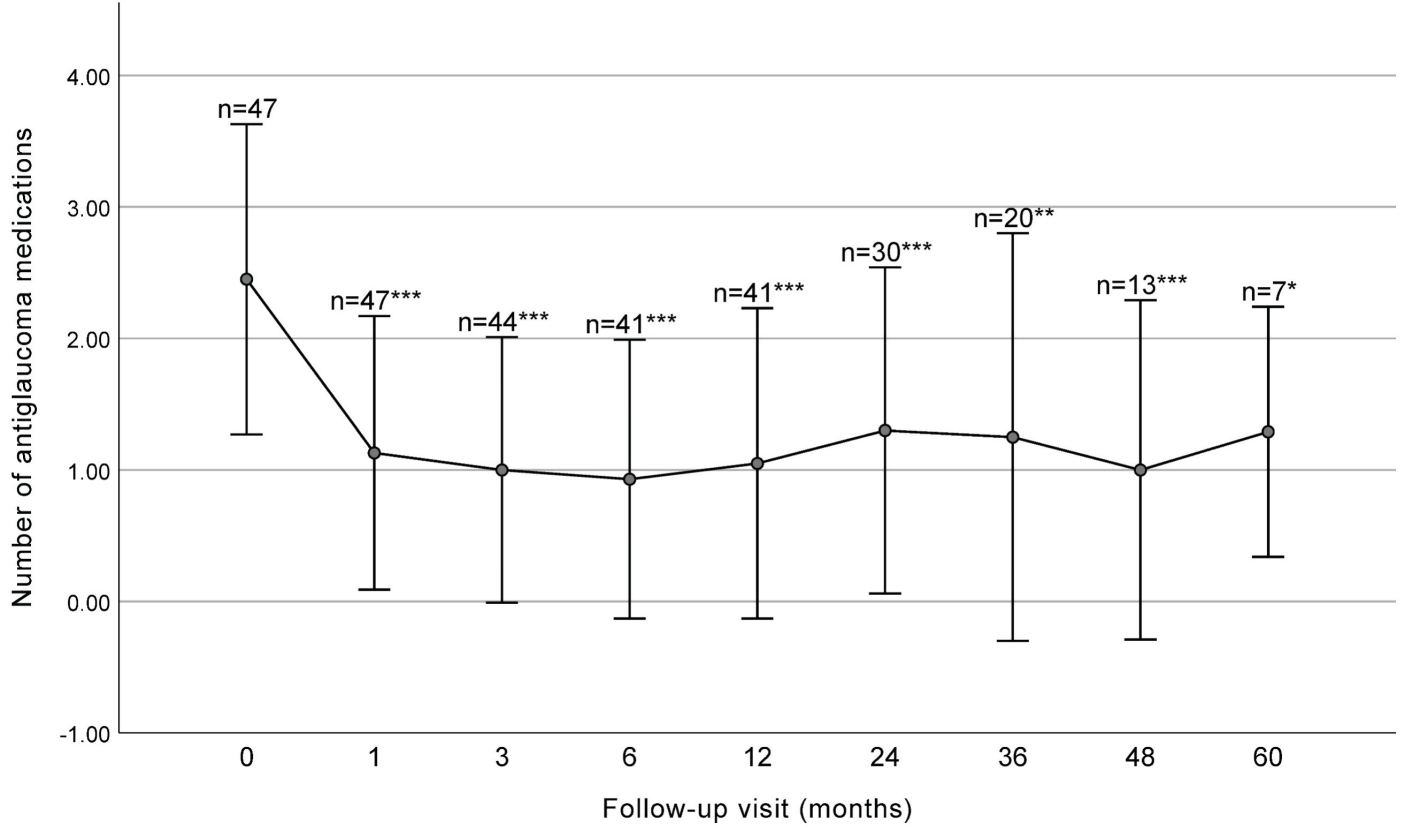
Number of antiglaucoma medications before surgery and at each follow-up visit (with SD bars). The number significantly decreased at each follow-up visit before 36 months. *** P< 0.001, ** P < 0.01, * P< 0.05.

The mean number of antiglaucoma medications significantly decreased from 2.45 ± 1.18 at baseline to 1.30 ± 1.24 at 2 years (*P* < 0.001) and 1.25 ± 1.55 at 3 years (*P* = 0.001) postoperatively (Figure 2 and Table 2).

BCVA of all patients before surgery and at 1 month postoperatively is presented in Table 3. There was a significant improvement in the average BCVA at 1 month after surgery (from 0.39 ± 0.29 to 0.48 ± 0.34) (*P* = 0.005).

The number of PAS quadrants reported by gonioscopy and UBM before and after surgery is presented in Table 4. A statistically significant decrease in the number of PAS quadrants was identified through a paired t-test when comparing baseline measurements to those obtained from patients who underwent both gonioscopy and UBM examinations at intervals ranging from 1 month to 36 months post-surgery. There was no statistically significant difference in PAS quadrants between consecutive follow-up visits, suggesting that PAS remained stable throughout the cohort following the surgical intervention. Besides, the variables associated with the decrease in IOP and the reduction of antiglaucoma medications 1 year after surgery were analyzed. As indicated in Table 5, the reduction of IOP showed a positive correlation with baseline IOP. Moreover, the reduction in antiglaucoma medications positively correlated with number of preoperative antiglaucoma medications used.

**Figure 3:**
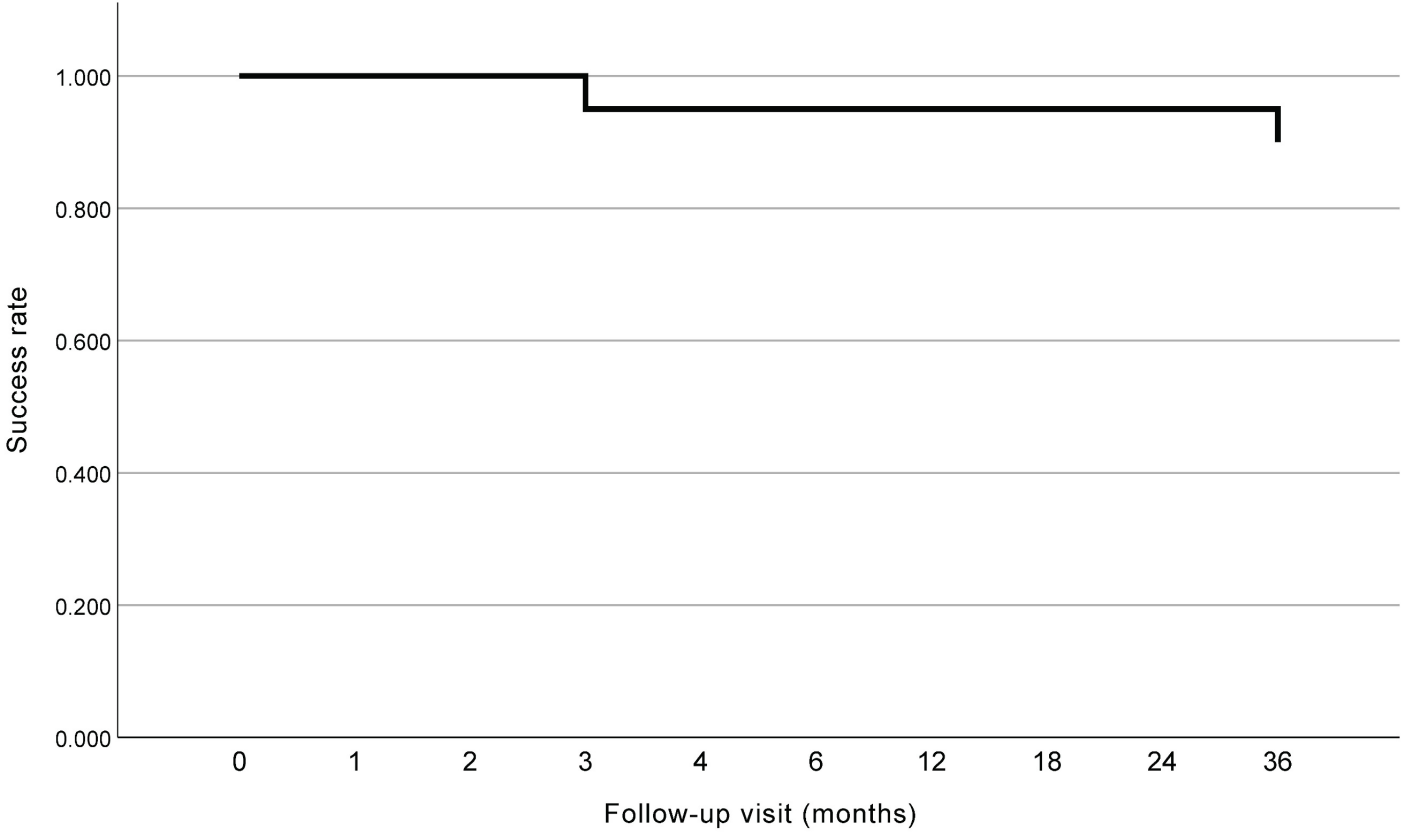
Kaplan-Meier curve of patients reached 3 years after surgery.

To evaluate the long-term success rate of AIT when combined with phacoemulsification, IOL implantation, and GSL in patients with PACG, we selected the patients who had completed 36 months of follow-up after surgery (n = 20). All these patients underwent GSL, Trabctome, phacoemulsification, and IOL implantation. The Kaplan-Meier curve is shown in Figure 3. The success rate of the surgery was 95.0% and 90.0% for patients with PACG at 2 years and 3 years postoperatively, respectively. Two patients experienced surgical failure at 3 months and 36 months postoperatively, respectively, and no secondary surgery was conducted.

All eyes presented with hyphema postoperatively due to reflux hemorrhage from SC, which was absorbed without additional treatment. Among the 47 eyes, elevated IOP within one month postoperatively was observed in 2 eyes (4.3%), which was alleviated by adjusting medication. Re-occurrence of PAS at the position of the removed TM tissue was observed in 3 eyes, which received peripheral argon laser iridoplasty as soon as the reformation of PAS was observed at the 1-month visit.

## Discussion

In this study, we demonstrated the long-term efficacy and safety of AIT combined with phacoemulsification, IOL implantation, and GSL in patients with PACG. Building upon our previous research 15, we believe this is the first study to investigate the 3-year outcomes of combined cataract surgery, GSL, and AIT procedures. Over the 3-year follow-up period, a consistent reduction in IOP and the number of required antiglaucoma medications was observed. BCVA improved significantly one month postoperatively compared to baseline. Additionally, PAS was effectively relieved over the long term after surgery.

The central feature of PACG is the closure of anterior chamber angle, which can lead to increased IOP and irreversible damage to the optic nerve. To prevent optic nerve damage, various treatment strategies for PACG have been developed to reduce IOP. Antiglaucoma medications, such as pilocarpine and brimonidine, can lower IOP and reopen the drainage angle 2. However, as the disease progresses, surgical intervention is often necessary. There is no single surgical approach that is universally accepted as the most effective. Cataract surgery, trabeculectomy, GSL, glaucoma implant, and cyclodestructive procedures have shown effectiveness in lowering IOP and preventing damage to the optic nerve 22.

Phacoemulsification combined with IOL implantation is the standard surgical procedure for cataract treatment. Previous studies have demonstrated that cataract surgery can reduce IOP and improve the VF in patients with PACG 23, 24. However, it is more effective in the early stages of PACG 25. The need for additional glaucoma treatment is also a concern 26. In this report, among the 20 patients who completed the 3-year follow-up, the success rate was 90.0% (18/20), with no patient requiring a second surgery. The reduction of IOP (5.84 mmHg versus 4.6 mmHg) and antiglaucoma medications (1.20 versus no significant reduction) was much greater than those reported for cataract surgery alone 26, supporting the effectiveness of angle opening and TM manipulation in treating patients with PACG.

Phaco-GSL (phacoemulsification and IOL implantation combined with GSL) is another combined surgery that is increasingly used in PACG. It has the potential to reduce IOP and the number of required antiglaucoma medications 27. However, some studies have reported that phaco-GSL does not significantly differ from cataract surgery in terms of IOP, requirements for antiglaucoma medication, or complication rates 10, 13, 28. Furthermore, Tian *et al.* compared the outcomes of phaco-GSL in patients with acute or chronic primary angle closure (PAC) /PACG and found a higher success rate in acute patients (100% versus 64.3%) 29. White *et al.* reported similar results 30. The disadvantages of phaco-GSL suggesting that the temporary re-opening of the angle achieved by GSL poses a risk of PAS recurrence in the long term. Moreover, significant changes in the architecture and arrangement of trabecular fibres, which are considered a basic pathological change in PACG 31, indicate that phaco-GSL alone is insufficient for reconstructing aqueous humor drainage. To identify a combined surgery that can sustainably reduce IOP and PAS in patients with chronic PACG, both the anterior chamber angle and TM should be considered.

AIT aims to remove the TM and the inner wall of SC through a minimally invasive internal approach. In Trabectome surgery, a strip of TM and the connected inner wall of SC are vaporized using high-frequency electrocautery energy. The KDB is a disposable gonioscopic ophthalmic knife designed to create parallel incisions in the TM, producing a strip of free tissue that can be removed using a gonioscopic approach 32. Both surgeries aim to enhance physiological outflow, thereby reducing IOP, and were initially used in POAG and secondary glaucoma. A previous study reported one-year follow-up outcomes of Trabectome surgery 15. Dorairaj *et al.* combined cataract surgery, GSL, and KDB-assisted trabeculectomy in patients with PACG and reported a reduction in IOP of 12.0 mmHg and a decrease in the number of medications by 1.7 per eye 17-19. However, the effectiveness of this surgery in Chinese patients with PACG has not been studied. The microhook ab interno trabeculotomy (μLOT) is an emerging MIGS procedure that uses a small metal hook to incise TM, facilitating the outflow of aqueous humor 33. Despite its small incisional cross‑sectional area, μLOT was reported to be as effective as KDB in controlling IOP and reducing medication usage 34. A multicenter study in China reported one-year outcomes of combined cataract surgery, GSL, and μLOT in PACG patients, observing an IOP reduction of 13.2 mmHg and a medication reduction of 1.7 medications 35. μLOT has the potential to offer a minimally invasive, effective alternative for reducing IOP and managing glaucoma in patients with PACG. Suture trabeculotomy (SLOT) ab interno is another potential MIGS technique, but it is currently primarily utilized in POAG and secondary glaucoma 36, 37.

In the current study, the effectiveness and safety of phacoemulsification and IOL implantation combined with GSL and AIT were demonstrated during a 3-year mean follow-up time. The results showed a sustained reduction in IOP and a decrease in the use of antiglaucoma medication for up to 60 months postoperatively. Compared to phaco-GSL, the combined surgery mentioned in this report showed similar decrease in IOP (6.35 mmHg versus 7.00 mmHg) and reduction in anti-glaucoma medication (1.40 versus 1.30) 13. However, as mentioned above, the long-term results of phaco-GSL surgery need further observation.

Phaco-trabeculectomy, a combination of phacoemulsification with IOL implantation and trabeculectomy, can effectively address TM blockage and has been evaluated as a treatment option for PACG. However, it is associated with more postoperative interventions and longer recovery times 11, 12, 28, 38. High incidence of complications, including shallow anterior chamber, choroidal detachment, hypotony maculopathy and blebitis, is also a problem to be considered 9. Our study found an equal reduction in IOP (5.84 mmHg) and a decrease in the number of antiglaucoma medications (1.20 medications) at 3 years compared to phaco-trabeculectomy at 5 years (5.69 mmHg and 1.9 medications) 38, but without any serious complications, unlike the 25 complications observed in 44 patients who underwent phaco-trabeculectomy. AIT removes a portion of the TM and SC via an internal approach, allowing aqueous humor to directly enter the SC, bypassing the blocked TM. GSL releases adhesions between the iris and the angle. The combination of AIT and GSL may effectively restore aqueous humor drainage in both the anterior chamber angle and the TM. Furthermore, the minimally invasive internal approach used in AIT causes less damage to the ocular structure than the external approach used in trabeculectomy, resulting in fewer complications.

Univariate linear regression analysis was conducted to assess the association between various variables and the reductions in IOP and the use of antiglaucoma medication. Similar to the results of our previous study, patients with higher preoperative IOP experienced greater IOP reduction, and those using more antiglaucoma medications before surgery experienced greater medication reduction. A possible explanation for these results is that some patients with higher IOP and more antiglaucoma medications may typically have a shorter disease duration and a lower likelihood of developing acute, severe PAS. Conversely, patients with a longer disease course, end-stage PAS, and severe TM disease may still benefit from combined phacoemulsification, IOL implantation, GSL, and AIT.

Several study limitations should be considered. First, all patients included in our study underwent phacoemulsification, IOL implantation, GSL, and AIT, and there was no control group. A randomized controlled trial comparing patients with PACG who underwent phacoemulsification, IOL implantation, and GSL with or without AIT would further demonstrate the efficacy of the procedure. Secondly, our center has only recently started using KDB surgery. As a result, there is no compelling comparison between KDB trabeculectomy and Trabectome. Additional data will be reported after a longer follow-up period.

In conclusion, the combination of phacoemulsification, IOL implantation, GSL, and AIT is a highly effective and safe surgical approach for reducing IOP and the number of required anti-glaucoma medications in patients with PACG during long-term follow-up. This approach may offer a less invasive option for patients with chronic and severe PAS.

## Figures and Tables

**Table 1:** Demographic and Glaucoma Status Data at Baseline.

Variables	Results
Gender, Male:Female	22:25
Age, mean years ± SD	68.26 ± 9.52
Follow-up time, mean months ± SD	40.96 ± 22.71
Preoperative BCVA, mean ± SD	0.40 ± 0.29
Preoperative IOP, mean mmHg ± SD	20.93 ± 6.53
Number of preoperative antiglaucoma drugs, mean ± SD	2.45 ± 1.18
Number of preoperative PAS quadrant, mean ± SD	3.49 ± 1.00
Visual field defect, mean MD ± SD	-22.16 ± 10.38
Combined surgery, Trabectome:trabeculectomy with KDB	40:7

Note: BCVA, best corrected visual acuity; IOP, intraocular pressure; PAS, peripheral anterior synechiae; KDB, Kahook dual blade; MD, mean deviation; SD, standard deviation.

**Table 2:** IOP and number of antiglaucoma medications at baseline and at each follow up visit.

Follow-up time point	n	IOP, mean mmHg ± SD	*P*	Number of medications, mean ± SD	*P*
Baseline	47	20.93 ± 6.53		2.45 ± 1.18	
1 month	47	14.18 ± 3.78	**< 0.001**	1.13 ± 1.04	**< 0.001**
3 months	44	13.57 ± 3.01	**< 0.001**	1.00 ± 1.01	**< 0.001**
6 months	41	14.00 ± 2.57	**< 0.001**	0.93 ± 1.06	**< 0.001**
12 months	41	14.58 ± 2.83	**< 0.001**	1.05 ± 1.18	**< 0.001**
24 months	30	14.78 ± 2.64	**< 0.001**	1.30 ± 1.24	**< 0.001**
36 months	20	15.09 ± 3.63	**< 0.001**	1.25 ± 1.55	**0.001**
48 months	13	14.50 ± 1.94	**0.003**	1.00 ± 1.29	**< 0.001**
60 months	7	13.50 ± 2.70	**0.006**	1.29 ± 0.95	**0.016**

Note: IOP, intraocular pressure; n, number; SD, standard deviation.

**Table 3:** Best corrected visual acuity before surgery and 1 month after surgery.

Follow-up time point	Number					BCVA	*P*
	< 0.1	0.1 - 0.3	0.3 - 0.5	0.5 - 1.0	≥ 1.0		
Baseline	10	8	7	20	2	0.39 ± 0.29	
1 month	8	6	6	20	7	0.48 ± 0.34	**0.005**

Note: BCVA, best correct visual acuity.

**Table 4:** Number of PAS quadrants in patients before and after surgery.

Follow-up time point	Number of PAS quadrants					*P*
	0	1	2	3	4	
Baseline	0	0	6	7	34	
1 month	8	8	10	9	4	**< 0.001**
3 months	8	7	11	7	4	**< 0.001**
6 months	6	10	7	7	4	**< 0.001**
12 months	8	10	5	7	4	**< 0.001**
24 months	4	9	3	4	5	**< 0.001**
36 months	3	7	1	1	4	**< 0.001**

Note: PAS, peripheral anterior synechiae.

**Table 5:** Univariate regression analysis for factors correlated with the reduction of IOP (R^2^ = 0.843) and reduction of the number of antiglaucoma medications (R^2^= 0.354) 1 years after surgery.

Variable	IOP		Number of medications	
	Univariate coefficient	*P*	Univariate coefficient	*P*
Age	0.068	0.171	0.015	0.453
Baseline IOP	0.950	**< 0.001**	-0.008	0.777
Baseline antiglaucoma medications	0.340	0.461	0.803	**< 0.001**
Baseline PAS quadrants	-0.177	0.695	0.081	0.671

Note: IOP, intraocular pressure; PAS, peripheral anterior synechiae.

## Abbreviations

AIT: Ab-interno trabeculectomy
BCVA: Best corrected visual acuity
GSL: goniosynechialysis
IOL: Intraocular lens
IOP: Intraocular pressure
KDB: Kahook Dual Blade
MIGS: Minimally invasive/ microinvasive glaucoma surgery
MD: Mean deviation
PAC: Primary angle closure
PACG: Primary angle-closure glaucoma
PAS: Peripheral anterior synechiae
POAG: Primary open-angle glaucoma
SC: Schlemm canal
SD: Standard deviation
SLOT: Suture trabeculotomy
TM: Trabecular meshwork
UBM: Ultrasound biomicroscopy
μLOT: Microhook ab interno trabeculotomy
VF: Visual field
